# The Role of Prenatal Psychosocial Stress in the Associations of a Proinflammatory Diet in Pregnancy With Child Adiposity and Growth Trajectories

**DOI:** 10.1001/jamanetworkopen.2022.51367

**Published:** 2023-01-20

**Authors:** Carmen Monthé-Drèze, Izzuddin M. Aris, Sheryl L. Rifas-Shiman, Nitin Shivappa, James R. Hebert, Emily Oken, Sarbattama Sen

**Affiliations:** 1 Department of Pediatric Newborn Medicine, Brigham and Women’s Hospital, Harvard Medical School, Boston, Massachusetts; 2 Division of Chronic Disease Research Across the Lifecourse, Department of Population Medicine, Harvard Medical School and Harvard Pilgrim Health Care Institute, Boston, Massachusetts; 3 South Carolina Statewide Cancer Prevention and Control Program and Department of Epidemiology and Biostatistics, University of South Carolina, Columbia; 4 Department of Nutrition, Connecting Health Innovations LLC, Columbia, South Carolina

## Abstract

**Importance:**

Prenatal psychosocial stress and nutrition may each program offspring adiposity, an important predictor of lifelong cardiometabolic health. Although increased stress and poor nutrition have been found to co-occur in pregnancy, little is known about their combined longitudinal associations in the offspring.

**Objective:**

To investigate whether the associations of the Dietary Inflammatory Index (DII) with offspring adiposity differ by prenatal stress levels and whether these associations change with age.

**Design, Setting, and Participants:**

Project Viva, a prospective prebirth cohort study of mother-child dyads in Massachusetts, included singleton children of mothers enrolled between April 1999 and July 2002, with follow-up visits at early childhood, midchildhood, and early adolescence. Data analysis was performed from October 31, 2020, to October 31, 2022.

**Exposures:**

Food frequency–derived DII score in pregnancy was the exposure. Effect modifiers included stress-related measures in pregnancy; depressive symptoms assessed using the Edinburgh Postnatal Depression Scale (EPDS), dichotomized at scores greater than or equal to 13 vs less than 13; and census tract–level social vulnerability (overall Social Vulnerability Index and its 4 main subindices), dichotomized at the 75th percentile.

**Main Outcomes and Measures:**

Overall adiposity, comprising sex- and age-standardized body mass index (BMI *z*), sum of subscapular and triceps skinfolds, fat mass index (FMI), and body fat percentage estimated using bioelectrical impedance analysis (BIA) and dual x-ray absorptiometry (DXA); and central adiposity, comprising waist circumference, ratio of subscapular to triceps skinfolds, and DXA–derived trunk FMI.

**Results:**

Among 1060 mother-child dyads, mean (SD) maternal age was 32.6 (4.6) years, and 811 (77%) mothers were non-Hispanic White. Mean (SD) DII score was –2.7 (1.3) units, Social Vulnerability Index level was 38th (27th) percentile, and 8% of mothers had depressive symptoms. Mean (SD) age of the children was 3.3 (0.3) years at the early childhood visit, 7.9 (0.8) years at the midchildhood visit, and 13.2 (0.9) years at the early adolescence visit. In adjusted analyses, children born to mothers in the highest (vs lowest) quartile of DII had slower decrease in BMI *z* scores (β, 0.03 SD units/y; 95% CI, 0.01-0.05 SD units/y), and faster adiposity gain (eg, BIA total FMI β, 0.11 kg/m^2^/y; 95% CI, 0.03-0.19 kg/m^2^/y) over time. Associations of prenatal DII quartiles with childhood adiposity were stronger (eg, BIA total FMI quartile 4 vs quartile 1 change in β, 1.40 kg/m^2^; 95% CI, 0.21-2.59 kg/m^2^) among children of mothers with high vs low EPDS scores in pregnancy, although EPDS scores did not modify the change over time. Associations of prenatal DII with adiposity change over time, however, were greater among children whose mothers lived in neighborhoods with a high (BIA percentage body fat: β, 0.55% per year; 95% CI, 0.04%-1.07% per year) vs low (β, 0.13% per year; 95% CI, –0.20 to 0.46% per year), percentage of racial and ethnic minorities, and residents with limited English-language proficiency.

**Conclusions and Relevance:**

The findings of this cohort study suggest that it may be useful to simultaneously evaluate prenatal diet and psychosocial stress in women as targets for interventions intended to prevent excess childhood adiposity.

## Key Points

**Question:**

Do associations between prenatal dietary inflammation and child adiposity trajectories differ by levels of psychosocial stress in pregnancy?

**Findings:**

In this cohort study of 1060 mother-child dyads, children exposed to the highest vs lowest quartile of diet-associated inflammation in utero experienced faster adiposity accrual from childhood to adolescence. Maternal depressive symptoms and social vulnerabilities during pregnancy strengthened these associations.

**Meaning:**

The findings of this study suggest that children exposed to poor prenatal dietary quality with high inflammatory potential in the presence of psychosocial stressors in utero may sustain worse adiposity outcomes lasting until adolescence.

## Teaser

This cohort study examines the association of maternal prenatal diet and stress indicators with adiposity in children.

## Introduction

Excessive childhood adiposity is associated with insulin resistance in childhood and subsequent risk for metabolic syndrome in adulthood.^1,2,3^ Therefore, an understanding of the early-life determinants of body composition could potentially inform strategies to improve cardiometabolic health outcomes later in life. Pregnancy is a sensitive life stage for developmental programming of offspring obesity and cardiometabolic health.^4^ We^5^ and others^6,7^ have previously noted that exposure to a proinflammatory diet during pregnancy is associated with higher child body mass index (BMI) and increased risk for childhood obesity. Most studies, however, have focused on single outcome time points, short-term adiposity outcomes in early childhood, or proxy measures for adiposity (ie, World Health Organization age- and sex-standardized *z* scores [BMI *z*]), and data on long-term adiposity into adolescence are lacking.^6,8,9,10,11,12^ Thus, it is unknown whether associations between maternal diet-associated inflammation and offspring adiposity vary as children age. Furthermore, although interventions to decrease diet-associated inflammation in pregnancy have been reported to improve offspring metabolic outcomes in animals,^13^ nutritional and lifestyle interventions in human pregnancy have not shown significant offspring obesity outcomes in early childhood, perhaps in part due to an incomplete understanding of factors that modulate the maternal diet–offspring adiposity relationship.^14,15,16^

Psychosocial stress in pregnancy has been shown to exacerbate the effects of toxic exposures and poor nutrition in pregnancy on offspring asthma and neurodevelopmental outcomes, suggesting that stress has an important moderating role on the outcome of prenatal exposures in the offspring.^17,18,19,20^ Although stress (eg, stressor exposure, such as social adversity, or the psychological response to stressors such as anxiety or depression) and poor diet quality are frequently studied in pregnancy, they have generally been evaluated separately for their associations with maternal and child health outcomes even though it is known that they often co-occur.^21,22^ For example, exposure to social adversity not only hinders access to health care systems but also reduces access to healthy foods, thus increasing food insecurity and risk for poor nutrition and subsequently increasing susceptibility to poorer health outcomes. However, limited data exist on the extent to which exposure to social adversity can modify the associations of dietary quality in pregnancy with offspring adiposity. To our knowledge, no published human studies have investigated their combined associations on offspring body composition.

To address these knowledge gaps, we investigated the longitudinal associations of the Dietary Inflammatory Index (DII) in pregnancy with growth and adiposity from childhood to early adolescence and examined the extent to which prenatal psychosocial stress may modify these associations. We hypothesized that higher DII scores during pregnancy would be associated with higher levels of adiposity in childhood and faster adiposity accrual from childhood through adolescence. We also hypothesized that children born to mothers who experienced higher levels of depressive symptoms and lived in neighborhoods with greater adversity during pregnancy would be most susceptible to prenatal diet–associated inflammation.

## Methods

### Study Design and Participants

This study population consisted of participants in Project Viva, an ongoing prospective cohort study of prenatal and perinatal influences on maternal and child health, as detailed elsewhere.^23^ Project Viva enrolled 2128 participants between April 1999 and July 2002, and study visits were conducted during pregnancy, childhood, and adolescence (eMethods in Supplement 1). Mothers provided written informed consent, and children provided verbal assent at follow-up visits; participants received financial compensation. The institutional review boards of Harvard Pilgrim Health Care Institute and the Brigham and Women’s Hospital approved the project in line with ethical standards established by the Declaration of Helsinki.^24^ We performed the data analysis from October 31, 2020, to October 31, 2022. This study followed the Strengthening the Reporting of Observational Studies in Epidemiology (STROBE) reporting guideline.

### Exposure: Dietary Inflammation in Pregnancy

Given prior work showing that prenatal DII scores in pregnancy are associated with offspring growth in early life, we explored the role of dietary inflammation in pregnancy on offspring adiposity into adolescence for the present study.^25,26^ The DII is a validated population-based measure that was developed to characterize and quantify the cumulative inflammatory potential of an individual’s diet.^27^ A detailed procedure for DII estimations in this cohort has been described previously.^28^ We derived the DII from semiquantitative food frequency questionnaires administered at the first (median, 9.9 weeks of gestation) and second (median, 27.9 weeks of gestation) study visits (eMethods in Supplement 1).

### Outcomes: Offspring Adiposity Indices

During research visits in early childhood (mean [SD] age, 3.3 (0.3) years), midchildhood (mean [SD] age, 7.9 [0.8] years), and early adolescence (mean [SD] age, 13.2 [0.9] years), research assistants measured weight, length/height, subscapular (SS) and triceps (TR) skinfold thicknesses, and waist circumference (WC), using standardized protocols.^23^ We determined BMI (calculated as weight in kilograms divided by height in meters squared) and derived BMI *z* scores using the World Health Organization child growth standards.^29^ We calculated the sum (SS + TR) of the 2 skinfold thicknesses to estimate overall adiposity and the ratio (SS/TR) to estimate central adiposity consistent with prior work and other studies of adiposity in young children.^30,31,32^ We used WC as a measure of central adiposity. At midchildhood and early adolescent visits only, we measured body composition using bioelectrical impedance (BIA) and whole-body dual x-ray absorptiometry (DXA), as detailed previously.^33^ We derived percentage body fat and fat mass index (FMI; calculated as fat mass in kilograms divided by height in meters squared) as measures of overall adiposity, and DXA-derived trunk FMI as a measure of central adiposity.

### Effect Modifiers: Psychosocial Stress

#### Maternal Depressive Symptoms

Mothers reported their depressive symptoms using the Edinburgh Postpartum Depression Scale (EPDS) at their second in-person study visit. The EPDS does not provide a clinical diagnosis of depression, but a score greater than or equal 13 indicates a positive screen, with a sensitivity of 65% and a specificity of 95%, for the diagnosis of depression.^34^ The EPDS was initially made for postpartum depression but has been validated and frequently used to evaluate prenatal depressive symptoms.^35,36^ In this study, we conceptualized depressive symptoms as a maladaptive psychological response to stress.^37,38^

#### Social Vulnerability Index

We used each participant’s residential address in early pregnancy to calculate the Social Vulnerability Index (SVI), a measure developed by the Centers for Disease Control and Prevention to identify at-risk populations vulnerable to stressors such as health emergencies.^39,40^ As detailed previously, the SVI is derived from a set of 15 US census community-level factors divided into 4 themes: socioeconomic status, household composition and disability, racial and ethnic minority and language status, and housing and transportation type.^41,42^ We conceptualized these factors as neighborhood-based chronic stressors that may play a critical role in creating vulnerability to dietary exposure in pregnancy (eMethods in Supplement 1). We used the overall and individual themes ranking (from 0 [lowest] to 100 [highest] vulnerability) as indicators for social vulnerability, and based on prior literature, we considered the top quartile to be the most vulnerable.^43,44,45^

### Statistical Analysis

We used the mean of the first and second trimester DII scores as our exposure because these were highly correlated (*r* = 0.61; *P* < .001) and because we did not seek to explore specific sensitive periods during pregnancy. We categorized DII scores into quartiles and used the lowest quartile of DII (Q1) as the reference category. We used the χ^2^ and analysis of variance tests for unadjusted associations by quartile of DII. We used linear mixed-effects models to examine longitudinal associations of prenatal DII with repeated adiposity measures from early childhood to early adolescence. We included DII quartiles and their interaction with child age (DII × age) as fixed effects and specified random effects for the intercept and linear slope using an unstructured covariance matrix to account for repeated observations in each child and reflect the heterogeneity in the data. In all models, we adjusted for the following confounders: child sex (except for models estimating BMI *z*), maternal age, educational level, race and ethnicity, parity, household income, smoking, and prepregnancy BMI (eMethods in Supplement 1). Race and ethnicity, as socially constructed variables, were included because individual and structural racism likely affect child health outcomes. Mothers reported their race and ethnicity (from the following options: non-Hispanic White, non-Hispanic Black, Hispanic, Asian, and others) via interviews and questionnaires at enrollment. If a participant chose more than 1 racial or ethnic group, we classified them in the other category, which also included American Indian or Alaskan Native.

We investigated effect modification by maternal depressive symptoms scores (EPDS≥13 vs <13 points) and SVI status (≥75th percentile [most vulnerable] vs <75th percentile, based on internal distribution of the Project Viva cohort) on the associations of prenatal DII with child adiposity from early childhood through early adolescence. We added multiplicative interaction terms into the fully adjusted models to evaluate the extent to which associations of prenatal DII scores with childhood adiposity were moderated by stress measures (prenatal DII × stress) and associations of prenatal DII scores with change in adiposity over time, or adiposity accrual, were moderated by stress measures (prenatal DII × age × stress).

We additionally conducted sensitivity analyses by repeating all analyses with our primary outcome (BMI *z* score), using imputed data sets for dyads with missing covariates. We performed all statistical analyses using Stata/SE, version 16.1 (StataCorp LLC) and defined statistical significance as α = .05.

## Results

### Participant Characteristics

Among 1060 included participants (eResults and eFigure 1 in Supplement 1), mean (SD) maternal age was 32.6 (4.6) years and prepregnancy BMI was 24.4 (4.9); 811 mothers (77%) were non-Hispanic White (Table 1). Mean (SD) DII score was –2.7 (1.3) units, Social Vulnerability Index level was 38th (27th) percentile, and 8% of mothers had depressive symptoms. Women with the lowest vs highest DII quartile were older, had lower prepregnancy BMI, were more likely to have a college education and identify as non-Hispanic White, and were less likely to belong to the top quartile of SVI. There were no significant differences in the proportion of women with a positive EPDS screen by DII quartile. eTable 1 in Supplement 1 describes the characteristics of participants included vs those excluded from the analyses.

**Table 1.** Characteristics of the 1060 Mother-Child Dyads Included by Quartile of Prenatal Dietary Inflammatory Index in Project Viva, a Cohort Recruited From the Boston, Massachusetts, Area in 1999-2002^a^

Characteristic	Total (N = 1060)	DII quartile (average first and second trimesters)				
		1 (n = 289)	2 (n = 28)0	3 (n = 271)	4 (n = 220)	*P* value
Maternal characteristics						
Age, mean (SD), y	32.6 (4.6)	33.4 (4.3)	33.5 (4.3)	32.2 (4.7)	30.9 (4.6)	<.001
DII, mean (SD units)	–2.7 (1.3)	–4.1 (0.3)	–3.2 (0.2)	–2.4 (0.3)	–0.7 (1.1)	<.001
Prepregnancy BMI, mean (SD)	24.4 (4.9)	23.5 (4.4)	23.9 (4.4)	24.9 (4.9)	25.7 (5.8)	<.001
Education, No. (%)						
Not a college graduate	268 (25)	40 (14)	61 (22)	72 (27)	95 (43)	<.001
College graduate	792 (75)	249 (86)	219 (78)	199 (73)	125 (57)	
Race and ethnicity, No (%)						
Asian or Pacific Islander	54 (5)	23 (8)	7 (2)	14 (5)	10 (5)	<.001
Hispanic	51 (5)	12 (4)	8 (3)	11 (4)	20 (9)	
Non-Hispanic Black	106 (10)	27 (9)	10 (4)	34 (13)	35 (16)	
Non-Hispanic White	811 (77)	217 (75)	249 (89)	204 (75)	141 (64)	
Other^b^	38 (3)	10 (3)	6 (2)	8 (3)	14 (6)	
Nulliparous, No. (%)						
No	540 (51)	123 (43)	134 (48)	155 (57)	128 (58)	<.001
Yes	520 (49)	166 (57)	146 (52)	116 (43)	92 (42)	
Household income >$70 000/y, No. (%)						
No	369 (35)	94 (33)	68 (24)	104 (38)	103 (47)	<.001
Yes	691 (65)	195 (67)	212 (76)	167 (62)	117 (53)	
Pregnancy smoking status, No. (%)						
Never	724 (68)	210 (73)	192 (69)	181 (67)	141 (64)	.004
Former	233 (22)	66 (23)	65 (23)	55 (20)	47 (21)	
Smoked during pregnancy	103 (10)	13 (4)	23 (8)	35 (13)	32 (15)	
SVI						
Overall percentile ranking, mean (SD), percentile	38 (27)	38 (27)	30 (22)	40 (27)	46 (31)	<.001
Top quartile, No. (%)						
No	831 (78)	224 (78)	249 (89)	212 (78)	146 (66)	<.001
Yes	229 (22)	65 (22)	31 (11)	59 (22)	74 (34)	
Top quartile in socioeconomic status subindex, No. (%)						
No	795 (75)	214 (74)	233 (83)	206 (76)	142 (65)	<.001
Yes	265 (25)	75 (26)	47 (17)	65 (24)	78 (35)	
Top quartile in household composition and disability subindex, No. (%)						
No	798 (75)	224 (78)	242 (86)	192 (71)	140 (64)	<.001
Yes	262 (25)	65 (22)	38 (14)	79 (29)	80 (36)	
Top quartile in racial and ethnic minority and language status subindex, No (%)						
No	797 (75)	227 (79)	236 (84)	191 (70)	143 (65)	<.001
Yes	263 (25)	62 (21)	44 (16)	80 (30)	77 (35)	
Top quartile in housing and transportation type subindex, No (%)						
No	795 (75)	219 (76)	219 (78)	198 (73)	159 (72)	.38
Yes	265 (25)	70 (24)	61 (22)	73 (27)	61 (28)	
Depressive symptoms, No. (%)						
No (EPDS <13)	972 (92)	264 (91)	261 (93)	250 (92)	197 (90)	.51
Yes (EPDS ≥13)	88 (8)	25 (9)	19 (7)	21 (8)	23 (10)	
Child characteristics						
Sex, No. (%)						
Male	520 (49)	131 (45)	137 (49)	149 (55)	103 (47)	.12
Female	540 (51)	158 (55)	143 (51)	122 (45)	117 (53)	

Abbreviations: BMI, body mass index (calculated as weight in kilograms divided by height in meters squared); DII, Dietary Inflammatory Index; EPDS, Edinburgh Postnatal Depression Scale; SVI, Social Vulnerability Index.

^a^ Values for categorical variables were compared using the χ^2^ test and continuous variables were compared using the analysis of variance test.

^b^ Other categories included American Indian or Alaska Native and more than 1 race and ethnicity.

### Associations of Prenatal DII Score With Childhood Adiposity and Adiposity Change

#### Childhood Adiposity

**Figure 1.**
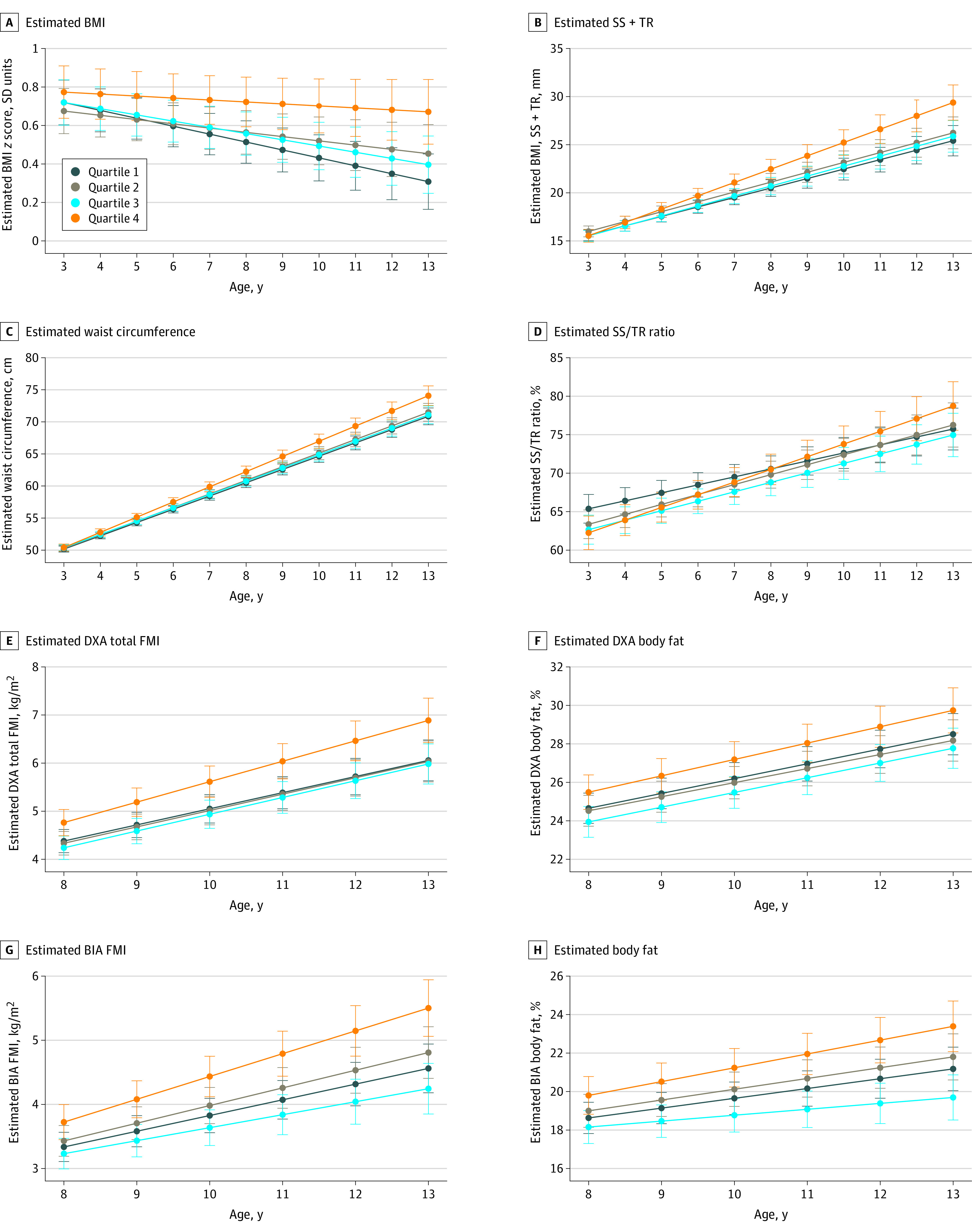
Estimated Trajectories of Child Adiposity Measures From Childhood Through Early Adolescence by Quartile of Prenatal Dietary Inflammatory Index Quartile Among Participants in Project Viva, a Cohort Recruited From the Boston, Massachusetts, Area in 1999-2002 Results are displayed as estimated means with 95% CIs from linear mixed model analyses adjusted for confounders and including a diet × age interaction term. A, World Health Organization age- and sex-standardized body mass index (BMI) (calculated as weight in kilograms divided by height in meters squared) *z* score. B, Subscapular skinfold (SS) + triceps skinfold (TR). C, Waist circumference. D, SS/TR ratio. E, Dual x-ray absorptiometry (DXA) total fat mass index (FMI). F, DXA body fat. G, Bioimpedance analyses (BIA) FMI. H, Estimated BIA body fat.

In unadjusted analysis, child adiposity measures differed across quartiles of prenatal DII scores (eTable 2 in Supplement 1). In adjusted models, there were no significant associations between prenatal DII scores and adiposity in early childhood (eg, BMI *z*: β, 0.05 SD units; 95% CI, –0.13 to 0.24 SD units) (Figure 1).

#### Adiposity Change (Accrual) Over Time

From childhood to early adolescence, offspring born to mothers in the highest vs lowest prenatal DII quartile had slower decrease in BMI *z* scores (β, 0.03 SD units/y; 95% CI, 0.01-0.05 SD units), and faster overall (eg, BIA FMI: β, 0.11 kg/m^2^/y; 95% CI, 0.03-0.19 kg/m^2^/y) and central (eg, WC: β, 0.30 cm/y; 95% CI, 0.10-0.50 cm/y) adiposity gain over time (Figure 1), resulting in higher BMI *z* scores (β, 0.36 SD units; 95% CI, 0.14-0.59 SD units) and adiposity in early adolescence (eTable 3 in Supplement 1). Estimates were higher for total (total FMI) vs central (trunk FMI) adiposity indices (eTable 3 in Supplement 1). Similar results were observed with DXA- and skinfold-based measures. Associations were null for both BIA and DXA measures of percentage of body fat.

### Effect Modification by Maternal EPDS Status in Pregnancy

#### Childhood Adiposity

Among included participants, 88 mothers (8%) had a positive EPDS screen characterized by higher EPDS scores (≥13). Associations of prenatal DII level with most measures of childhood adiposity (overall: eg, DXA total FMI: quartile 4 [Q4] vs Q1 change in β, 1.73 kg/m^2^; 95% CI, 0.52-2.95 kg/m^2^; central adiposity: eg, DXA trunk FMI: Q4 vs Q1 change in β, 0.77 kg/m^2^; 95% CI, 0.23-1.32 kg/m^2^) were stronger among offspring of mothers with EPDS scores greater than or equal to 13 in pregnancy. Interactions of DII × EPDS for DXA and BIA FMI as well as WC and DXA trunk FMI were all statistically significant (Table 2). Interaction analyses showed similar patterns with most proxy (anthropometry-based) measures of adiposity in childhood, although the 95% CIs included the null.

**Table 2.** Joint Associations of Prenatal DII and EPDS During Pregnancy With Adiposity Indices and Interactions With Child Age, Among Mother-Child Dyads in Project Viva^a^

Variable, by DII Q	EPDS score category	β (95% CI)	
		DII × EPDS interaction	DII × age × EPDS interaction
Anthropometry measures			
Overall adiposity			
BMI *z*, SD units (n = 1055)			
Q1	Low	1 [Reference]	1 [Reference]
Q2	High	0.21 (–0.40 to 0.82)	0.01 (–0.07 to 0.08)
Q3	High	0.33 (–0.26 to 0.92)	–0.03 (–0.10 to 0.05)
Q4	High	0.44 (–0.16 to 1.04)	–0.05 (–0.12 to 0.03)
*P* value for trend^b^	NA	.13	.19
SS + TR, mm (n = 1049)			
Q1	Low	1 [Reference]	1 [Reference]
Q2	High	0.46 (–2.52 to 3.43)	–0.19 (–1.08 to 0.70)
Q3	High	1.43 (–1.41 to 4.27)	–0.31 (–1.16 to 0.54)
Q4	High	2.40 (–0.55 to 5.36)	–0.13 (–0.98 to 0.72)
*P* value for trend^b^	NA	.08	.70
Central adiposity			
WC, cm (n = 1054)			
Q1	Low	1 [Reference]	1 [Reference]
Q2	High	0.88 (–1.58 to 3.33)	–0.14 (–0.86 to 0.58)
Q3	High	1.54 (–0.84 to 3.91)	–0.22 (–0.91 to 0.46)
Q4	High	3.14 (0.70 to 5.57)	–0.13 (–0.81 to 0.55)
*P* value for trend^b^	NA	.01	.69
SS/TR, % (n = 1049)			
Q1	Low	1 [Reference]	1 [Reference]
Q2	High	1.95 (–7.80 to 11.7)	–0.77 (–2.50 to 0.97)
Q3	High	–0.09 (–9.41 to 9.23)	–0.05 (–1.71 to 1.61)
Q4	High	–4.34 (–14.0 to 5.32)	–0.34 (–2.03 to 1.35)
*P* value for trend^b^	NA	.37	.87
Body composition measures			
Overall adiposity			
BIA FMI, kg/m^2^ (n = 908)			
Q1	Low	1 [Reference]	1 [Reference]
Q2	High	–0.06 (–1.30 to 1.18)	–0.03 (–0.32 to 0.26)
Q3	High	–0.17 (–1.35 to 1.02)	–0.03 (–0.32 to 0.25)
Q4	High	1.40 (0.21 to 2.59)	–0.10 (–0.38 to 0.19)
*P* value for trend^b^	NA	.05	.59
BIA fat % (n = 908)			
Q1	Low	1 [Reference]	1 [Reference]
Q2	High	–0.17 (–4.60 to 4.26)	–0.24 (–1.19 to 0.72)
Q3	High	–0.33 (–4.58 to 3.93)	–0.23 (–1.18 to 0.72)
Q4	High	3.08 (–1.19 to 7.36)	–0.83 (–1.79 to 0.14)
*P* value for trend^b^	NA	.22	.14
DXA FMI, kg/m^2^ (n = 704)			
Q1	Low	1 [Reference]	1 [Reference]
Q2	High	–0.14 (–1.49 to 1.21)	0.21 (–0.14 to 0.56)
Q3	High	–0.11 (–1.29 to 1.06)	0.07 (–0.20 to 0.35)
Q4	High	1.73 (0.52 to 2.95)	–0.18 (–0.48 to 0.12)
*P* value for trend^b^	NA	.03	.44
DXA fat % (n = 704)			
Q1	Low	1 [Reference]	1 [Reference]
Q2	High	–0.82 (–5.25 to 3.61)	0.17 (–0.77 to 1.11)
Q3	High	–0.37 (–4.22 to 3.48)	0.25 (–0.48 to 0.97)
Q4	High	3.50 (–0.51 to 7.52)	–0.96 (–1.77 to –0.14)
*P* value for trend^b^	NA	.20	.12
Central adiposity			
DXA trunk FMI, kg/m^2^ (n = 704)			
Q1	Low	1 [Reference]	1 [Reference]
Q2	High	–0.10 (–0.70 to 0.49)	0.13 (–0.05 to 0.30)
Q3	High	–0.04 (–0.56 to 0.48)	0.03 (–0.11 to 0.17)
Q4	High	0.77 (0.23 to 1.32)	–0.05 (–0.20 to 0.10)
*P* value for trend^b^	NA	.03	.75

Abbreviations: BIA, bioelectrical impedance analysis; BMI *z*, World Health Organization age- and sex-standardized body mass index score; DII, dietary inflammatory index; DXA, dual x-ray absorptiometry; EPDS, Edinburgh Postpartum Depression Scale; FMI, fat mass index; NA, not applicable; Q, quartile; SS, subscapular skinfold; TR, triceps skinfold; WC, waist circumference.

^a^ Linear mixed-effects models were used to estimate joint associations of maternal DII and EPDS scores among mother-child dyads with child adiposity. Maternal DII quartile category and EPDS category (high vs low) were included as a fixed effect and as an interaction with age. Model also included DII × EPDS and DII × age × EPDS interaction terms. Model adjusted for child age and sex (except for the BMI *z* outcome), maternal age at enrollment, race and ethnicity, educational level, parity, household income, pregnancy smoking status, and prepregnancy BMI.

^b^ *P* value for trend obtained from linear mixed-effects models constructed as above using the 4-category DII variable as a continuous variable.

#### Adiposity Change (Accrual) Over Time

Prenatal depressive symptoms did not modify associations of prenatal DII with adiposity change over time (*P* > .05 for interaction for DII × age × EPDS) (Table 2) for all measures (eg, DXA total FMI: Q4 vs Q1 change in β, –0.18 kg/m^2^/y; 95% CI, –0.48 to 0.12 kg/m^2^/y). Stratified analysis by EPDS category could not be conducted given the low numbers of participants with EPDS scores greater than or equal to 13.

### Effect Modification by Maternal SVI Status in Pregnancy

#### Childhood Adiposity

Prenatal SVI status, based on overall percentile ranking, moderated the associations of prenatal DII with direct (both BIA and DXA*; P < *.05 for interaction for DII × SVI) but not proxy (anthropometry-based) measures of adiposity in childhood (Table 3). For example, the magnitude of the associations of prenatal DII with DXA total FMI was stronger among children exposed to low (β, 0.60; 95% CI, 0.20-1.01) vs high (β, –0.13 kg/m^2^; 95% CI, –0.93 to 0.66 kg/m^2^/y) SVI in pregnancy (*P* = .03 for interaction for DII × SVI). Interaction analyses with subindices of the SVI showed that this was mainly associated with the household composition/disability subindex (eFigure 2 in Supplement 1, panel B) (*P* = .049 for interaction).

**Table 3.** Associations of DII Score in Pregnancy With Adiposity Indices Across Early Childhood, Midchildhood, and Early Adolescence Visits, and Interactions With Child Age Stratified by Maternal SVI Status During Pregnancy Among Mother-Child Dyads in Project Viva^a^

Variable	High SVI (>75th percentile)^b^		Low SVI (<75th percentile)^c^	
	β (95% CI)		β (95% CI)	
	Main effect	Diet × age interaction	Main effect	Diet × age interaction
**Anthropometric measures**				
Overall adiposity				
BMI *z*, SD units (n = 1055)				
Q1	1 [Reference]	1 [Reference]	1 [Reference]	1 [Reference]
Q2	0.39 (–0.07 to 0.84)	0.03 (–0.02 to 0.08)	–0.11 (–0.28 to 0.06)	0.12 (–0.09 to 0.32)
Q3	–0.25 (–0.64 to 0.13)	0.04 (–0.01 to 0.08)	0.05 (–0.14 to 0.23)	0.00 (–0.02 to 0.02)
Q4	–0.22 (–0.61 to 0.16)	0.04 (–0.00 to 0.08)	0.12 (–0.09 to 0.32)	0.03 (0.00–0.05)
*P* value for trend^d^	.13	.06	.13	.15
SS + TR, mm (n = 1049)				
Q1	1 [Reference]	1 [Reference]	1 [Reference]	1 [Reference]
Q2	1.29 (–0.86 to 3.44)	0.26 (–0.38 to 0.90)	0.25 (–0.61 to 1.11)	0.02 (–0.24 to 0.26)
Q3	–0.99 (–2.84 to 0.86)	0.08 (–0.45 to 0.61)	0.06 (–0.84 to 0.96)	0.04 (–0.22 to 0.29)
Q4	–1.24 (–3.12 to 0.64)	0.52 (0.02–1.02)	0.27 (–0.75 to 1.29)	0.34 (0.06– 0.63)
*P* value for trend^d^	.12	.07	.71	.04
Central adiposity				
WC, cm (n = 1054)				
Q1	1 [Reference]	1 [Reference]	1 [Reference]	1 [Reference]
Q2	1.94 (0.11 to 3.77)	0.21 (–0.31 to 0.73)	–0.24 (–0.94 to 0.47)	0.04 (–0.16 to 0.24)
Q3	–0.17 (–1.76 to 1.42)	0.01 (–0.42 to 0.45)	0.21 (–0.52 to 0.95)	–0.01 (–0.21 to 0.20)
Q4	0.06 (–1.54 to 1.66)	0.25 (–0.16 to 0.66)	0.21 (–0.63 to 1.04)	0.31 (0.08–0.54)
*P* value for trend^d^	.79	.32	.41	.04
SS/TR, % (n = 1049)				
Q1	1 [Reference]	1 [Reference]	1 [Reference]	1 [Reference]
Q2	1.50 (–5.35 to 8.35)	0.52 (–0.67 to 1.70)	–2.75 (–5.59 to 0.09)	0.19 (–0.31 to 0.70)
Q3	–2.89 (–8.73 to 2.95)	0.67 (–0.32 to 1.66)	–2.44 (–5.42 to 0.55)	0.06 (–0.46 to 0.57)
Q4	1.54 (–4.36 to 7.44)	0.86 (–0.08 to 1.80)	–4.54 (–7.90 to –1.17)	0.48 (–0.10 to 1.06)
*P* value for trend^d^	.85	.07	.01	.20
**Body composition measures**				
Overall adiposity				
BIA FMI (n = 908)				
Q1	1 [Reference]	1 [Reference]	1 [Reference]	1 [Reference]
Q2	0.56 (–0.38 to 1.50)	0.09 (–0.14 to 0.32)	–0.00 (–0.34 to 0.34)	0.02 (–0.061 to 0.10)
Q3	–0.28 (–1.08 to 0.52)	0.03 (–0.16 to 0.22)	–0.11 (–0.47 to 0.25)	–0.07 (–0.15 to 0.02)
Q4	0.16 (–0.63 to 0.94)	0.13 (–0.04 to 0.31)	0.50 (0.10 to 0.91)	0.10 (0.00 to 0.19)
* P* value for trend^d^	.98	.18	.08	.36
BIA fat % (n = 908)				
Q1	1 [Reference]	1 [Reference]	1 [Reference]	1 [Reference]
Q2	1.39 (–1.79 to 4.57)	0.09 (–0.62 to 0.80)	0.18 (–1.06 to 1.42)	0.03 (–0.25 to 0.31)
Q3	–1.50 (–4.17 to 1.18)	0.01 (–0.57 to 0.59)	–0.46 (–1.78 to 0.86)	–0.26 (–0.55 to 0.03)
Q4	–0.95 (–3.62 to 1.72)	0.30 (–0.25 to 0.85)	1.87 (0.39 to 3.35)	0.19 (–0.13 to 0.52)
* P* value for trend^d^	.32	.33	.08	.88
DXA FMI (n = 704)				
Q1	1 [Reference]	1 [Reference]	1 [Reference]	1 [Reference]
Q2	0.48 (–0.46 to 1.42)	–0.03 (–0.26 to 0.19)	–0.14 (–0.49 to 0.20)	0.00 (–0.08 to 0.09)
Q3	–0.35 (–1.17 to 0.48)	0.04 (–0.15 to 0.23)	–0.18 (–0.55 to 0.18)	0.00 (–0.08 to 0.09)
Q4	–0.13 (–0.93 to 0.66)	0.12 (–0.06 to 0.30)	0.60 (0.20 to 1.01)	0.08 (–0.02 to 0.18)
* P* value for trend^d^	.53	.15	.03	.19
DXA fat % (n = 704)				
Q1	1 [Reference]	1 [Reference]	1 [Reference]	1 [Reference])
Q2	1.22 (–1.66 to 4.10)	–0.20 (–0.75 to 0.36)	–0.39 (–1.56 to 0.78)	–0.05 (–0.29 to 0.18
Q3	–1.66 (–4.16 to 0.85)	0.05 (–0.42 to 0.52)	–0.78 (–2.03 to 0.47)	–0.04 (–0.28 to 0.20)
Q4	–0.97 (–3.41 to 1.46)	0.25 (–0.19 to 0.68)	1.53 (0.14 to 2.92)	0.03 (–0.24 to 0.30)
* P* value for trend^d^	.27	.19	.15	.88
Central adiposity				
DXA trunk FMI (n = 704)				
Q1	1 [Reference]	1 [Reference]	1 [Reference]	1 [Reference]
Q2	0.20 (–0.22 to 0.61)	–0.01 (–0.12 to 0.10)	–0.08 (–0.23 to 0.07)	0.01 (–0.04 to 0.05)
Q3	–0.14 (–0.50 to 0.22)	0.02 (–0.07 to 0.12)	–0.10 (–0.26 to 0.07)	0.00 (–0.05 to 0.04)
Q4	–0.05 (–0.40 to 0.30)	0.06 (–0.02 to 0.15)	0.24 (0.06 to 0.42)	0.04 (–0.01 to 0.09)
* P* value for trend^d^	.58	.13	.05	.19

Abbreviations: BIA, bioelectrical impedance analysis; BMI *z*, World Health Organization age- and sex-standardized body mass index score; DII, Dietary Inflammatory Index; DXA, dual-energy x-ray absorptiometry; FMI, fat mass index; Q, quartile; SS, subscapular skinfold; SVI, Social Vulnerability Index; TR, triceps skinfold thickness; WC, waist circumference.

^a^ Linear mixed-effects models were used to estimate mean differences in each adiposity measure by quartile of maternal DII score. Quartile of maternal DII category was included as a fixed effect and as an interaction with age and adjusted for child age and sex (except for the BMI *z* outcome), maternal age at enrollment, race and ethnicity, educational level, parity, household income, pregnancy smoking status, and prepregnancy BMI.

^b^ There were data for 228 participants for BMI *z, *SS + TR, and SS/TR; 229 participants for WC; 206 participants for BIA FMI and BIA fat %; and 180 participants for DXA FMI, DXA fat %, and DXA trunk FMI.

^c^ There were data for 827 participants for BMI *z*; 821 participants for SS + TR and SS/TR, %; 825 participants for WC; 702 participants for BIA FMI and BIA fat %; and 524 participants for DXA FMI, DXA fat %, and DXA trunk FMI.

^d^ *P* value for trend obtained from linear mixed-effects models constructed as above using the 4-category DII variable as a continuous variable.

#### Adiposity Change (Accrual) Over Time

**Figure 2.**
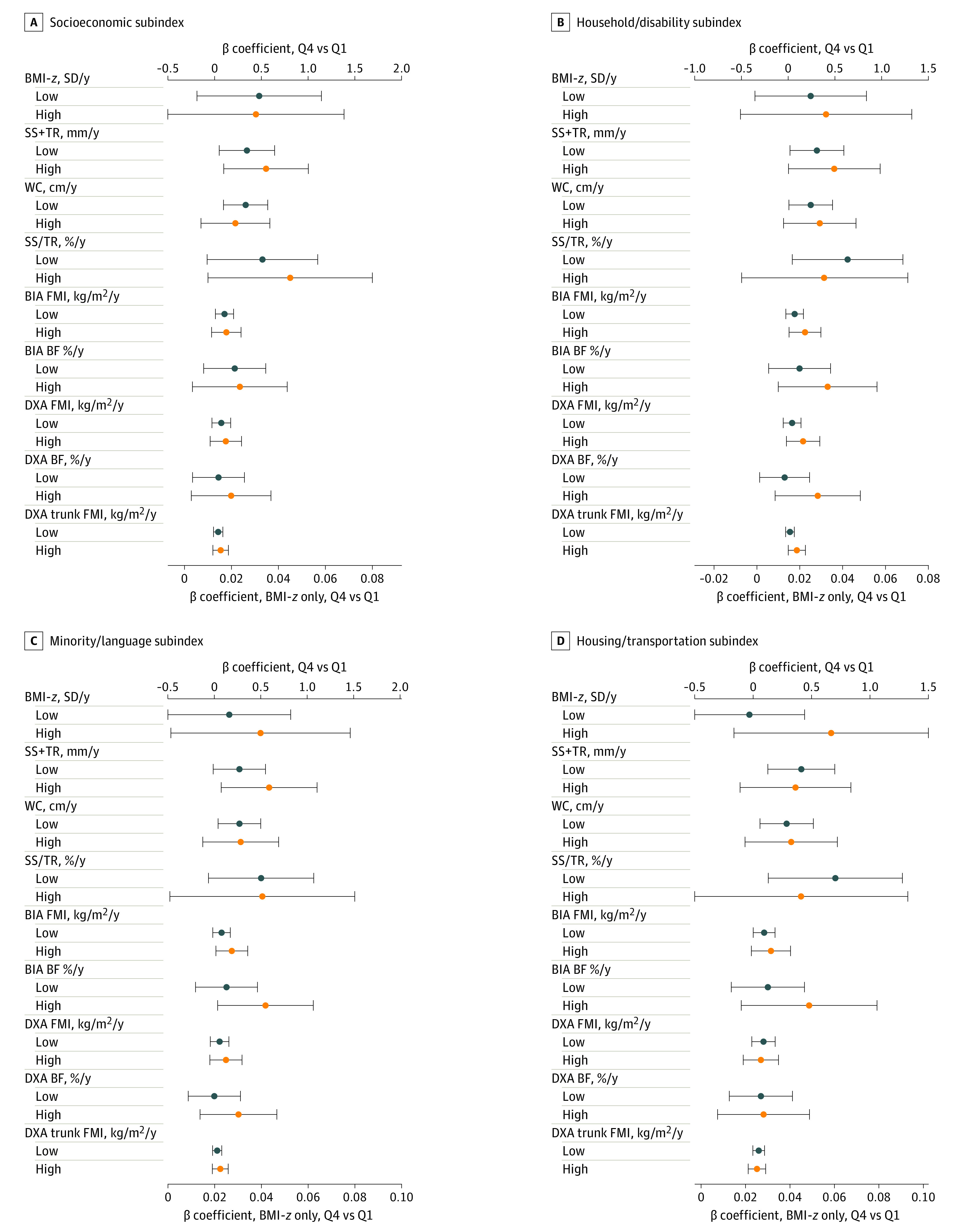
Prenatal Dietary Inflammatory Index (DII) Quartile (Q) and Adiposity Change Stratified by Each Subindex Theme of the Social Vulnerability Index (SVI) Results are β coefficients with 95% CIs for DII × age from linear mixed model analyses with prenatal DII quartile as a fixed effect and as an interaction with age, adjusted for child age and sex (except for the World Health Organization age- and sex-standardized body mass index [BMI]-*z* score outcome), confounders (maternal age at enrollment, race and ethnicity, educational level, parity, household income, pregnancy smoking status, and prepregnancy BMI), stratified by each SVI subindex category (high: >75th percentile; low: <75th percentile). BF indicates body fat percentage; BIA, bioimpedance analyses; DXA, dual x-ray absorptiometry; FMI, fat mass index; Q, quartile; SS, subscapular skinfold; TR, triceps skinfold; WC, waist circumference.

The association of prenatal DII with adiposity change did not differ significantly between children exposed in utero to high vs low overall SVI (Table 3). Interaction analyses with subindices of the SVI showed that associations of prenatal DII with change in BIA percentage body fat over time were strongest among children whose mothers lived in neighborhoods with a high (β, 0.55% per year; 95% CI, 0.04%-1.07% per year) vs low (β, 0.13% per year; 95% CI, –0.20% to 0.46% per year) percentage of racial and ethnic minority populations during pregnancy (Figure 2C). Similar results were observed with other adiposity measures (DXA percentage body fat, SS + TR), although the 95% CI included the null (Figure 2C). Sensitivity analyses with imputed data for the associations of prenatal DII with the primary outcome, BMI *z* score (eTable 4 in Supplement 1), and for effect modification by EPDS (eTable 5 in Supplement 1) and SVI (eTable 6 in Supplement 1) yielded results similar to those obtained from complete case analysis.

## Discussion

In this study, we observed that higher prenatal DII scores were associated with greater overall and central adiposity in early adolescence and faster adiposity gains from childhood to adolescence. Furthermore, maternal depressive symptoms strengthened associations of prenatal DII with childhood adiposity. In addition, the associations of prenatal DII with adiposity accrual were strongest among children whose mothers lived in neighborhoods with a high percentage of racial and ethnic minority populations and residents with limited English-language proficiency.

Our findings corroborate and extend prior studies that explored the associations of maternal dietary indices with offspring body composition. While several studies have been published on the associations of pregnancy diet quality and adiposity in early childhood, few have examined longitudinal associations to adolescence. We report that the associations between prenatal DII and offspring adiposity emerge in later childhood and early adolescence. For example, children exposed to the highest vs lowest quartile of DII in utero had a 0.09 kg/m^2^ greater yearly FMI gain, resulting in a 0.83 kg/m^2^ higher FMI in early adolescence. These findings are consistent with a previous study in this cohort that noted that maternal DII was associated with faster BMI *z* trajectories later in childhood, although this study did not evaluate direct measures of adiposity.^5^ In a pooled analysis of 7 European cohorts in the ALPHABET consortium, Chen et al^9^ reported that higher adherence to the Dietary Approaches to Stop Hypertension diet plan was associated with lower FMI in late (approximate age, 10 years), but not early (approximate age, 3 years) or mid (approximate age, 6 years) childhood, which is consistent with our results. These studies suggest that the programming effects of nutrition may not emerge until later childhood and early adolescence. Therefore, trials evaluating the effects of lower pregnancy dietary inflammation on offspring adiposity may require long-term follow-up at least through early adolescence.

It is biologically plausible that maternal diet–associated inflammation has an age-dependent influence on offspring adiposity. Maternal inflammation is associated with offspring adiposity and adipogenesis in both animal and human studies.^26,46^ Furthermore, maternal diet has been shown to induce transcriptomic and epigenetic alterations in the offspring that may not lead to adiposity until later childhood, when children are more directly and independently exposed to a more obesogenic environment.^47,48,49^ In addition, adipocyte proliferation is substantial during the first year of life and during adolescence, but remains low in between and plateaus after; in addition, adipocytes from children with obesity are larger than adipocytes from those without obesity starting in middle childhood, therefore highlighting these periods as most sensitive to in utero adipogenic exposures.^50,51,52^

Few animal experiments and, to our knowledge, no human studies have evaluated the combined or interactive longitudinal associations of nutrition and stress during pregnancy with offspring health.^17,18,19,20,22^ Most studies focusing on prenatal dietary interventions have not considered maternal stress, which may affect intervention outcomes due to poor motivation or altered metabolic pathways. Our results also suggest that stress is not a singular construct in which stressors of different types have the same influence on health outcomes.^37^ Herein, we noted that different types of pregnancy stress may differentially modulate associations between pregnancy exposures and offspring outcomes. Therefore, it is essential to use a more precise and unified conceptualization of pregnancy stress to gain a deeper understanding of its effect on offspring health.^37^

Underlying mechanisms mediating the interplay between nutrition and stress during pregnancy are not well understood. Evidence from studies in nonpregnant individuals noted substantial recursive, bidirectional associations among stress, dietary behavior, and nutritional biochemistry.^17,53^ Prenatal stress can prime the inflammatory response by inducing an exaggerated cytokine and metabolic response following an inflammatory/immune challenge, such as a poor diet, thereby enhancing its inflammatory potential.^54,55,56^ Animal and nonhuman primate studies also suggest that both prenatal stress exposure and maladaptive stress response may impact the development of childhood obesity through complex metabolic (ie, adipogenesis) and behavioral (ie, hyperphagia) pathways mediated in part by hyperactivity of the maternal hypothalamus-pituitary-adrenal axis, dysregulation of the fetal hypothalamus-pituitary-adrenal axis, and increased inflammation.^57,58,59,60,61^ Therefore, given the bidirectional associations between stress and diet, future studies are also needed to investigate the moderating role of diet in the associations of prenatal stress with child adiposity.

Social vulnerability has been reported to exacerbate health outcomes in maternal-child health studies.^41,42^ We observed that, as prenatal DII scores increased from the lowest to highest DII quartile, children of mothers who lived during their pregnancy in neighborhoods with a high proportion of racial and ethnic minority groups had a 0.55% body fat greater yearly adiposity gain. It is possible that factors such as structural racism and discrimination, which often result in inequitable access to education, wealth, employment, and health care, may play a role, as it has been previously reported in birth outcome disparities.^62,63^ Therefore race, a social construct, may serve as a surrogate for racism and health inequities. Furthermore, these societal factors together may lead to toxic environmental exposures and chronic psychosocial stress in pregnancy, further contributing to adverse fetal programming.^64^ Our results suggest that the SVI may be used to identify populations with the highest susceptibility to in utero adverse dietary exposures to inform targeted dietary interventions in pregnancy.

We also report that associations of prenatal DII with childhood adiposity were observed only among children whose mothers lived in neighborhoods with low percentages of elderly, youth, single-parent households, and individuals with disability, mirroring results based on overall SVI. Although this observation was unexpected, given that a low household composition SVI score is indicative of lower social vulnerability, it must be considered within the limitations of the SVI. Communities with low percentages of elderly, youth, single-parent households, and individuals with disability may be disadvantaged in other aspects of the neighborhood environment (eg, reduced access to health care services and/or healthy food choices) that are not adequately captured by the SVI and are also linked to disparate health behaviors and outcomes.^65,66^ These aspects of the built environment may enhance or protect against obesity. Nevertheless, further studies are warranted to clarify the role of household composition in the association of dietary quality in pregnancy with childhood obesity outcomes.

The clinical implications of our findings for long-term health are unclear given our small effect estimates. While we found that adolescent children exposed to the highest quartile of DII in utero have 0.36 SD units higher BMI *z* scores, others have reported that an increase in BMI *z* score by 0.5 SD units increases the risk for developing metabolic syndrome.^67^ Although our study found relatively small differences in adiposity among children exposed to DII during pregnancy, we found that these associations strengthened over time. Therefore, these differences may still have substantial health consequences during later life. However, further research is needed to evaluate associations with longer term cardiometabolic health outcomes.

### Strengths and Limitations

Our study has several strengths, including its relatively large sample size, and the quality and scope of data used in analyses. We also conducted sensitivity analyses with imputed data to assess the robustness of our findings. We included several confounders, including prepregnancy BMI, which is independently associated with offspring adiposity.^14,16^ We assessed direct measures of adiposity using DXA, and findings were similar to BIA measures. In addition, a common limitation of prior research exploring the role of prenatal stress has been the use of a singular measure of stress or the lack of precise language for describing stress measures. Stressors (eg, life events, neighborhood deprivation) and psychological or behavioral responses to those stressors (eg, perceived stress, anxiety, depression) capture different aspects of the human stress experience and may not be highly correlated.^37,68^ In this study, we assessed both dimensions of stress to help define the extent to which these stress processes interact with prenatal diet quality to shape offspring adiposity outcomes.

Despite its strengths, our study has limitations. First, we cannot exclude the possibility of residual confounding. Second, we did not evaluate the role of child diet as it may be on the causal pathway between prenatal DII and child adiposity. Mothers are likely to offer their children dietary choices similar to their own, and older children may model eating behaviors after their caregivers. In addition, high-fat or junk-food diet in pregnancy has been shown in animal models to program offspring food preferences and eating behaviors via alterations in neural appetite circuitries.^69,70^ Third, we did not adjust the DII for total energy intake, which could confound results. However, prior studies have found that prenatal DII remains a significant predictor of childhood adiposity even after energy adjustment and after accounting for child diet.^9^ Fourth, self-reported dietary data were used, which might increase nondifferential measurement errors that may bias results toward the null. However, nutrient intakes estimated from the food frequency questionnaires were comparable with biomarker concentrations of several nutrient measures in blood.^71^ Fifth, the pattern of study findings was mixed in that associations with direct measures of adiposity did not always follow the same patterns with indirect, anthropometry-based measures. Sixth, our results may have been biased toward the null due to being underpowered because the study population, compared with excluded participants, had lower SVI and was generally healthier, with lower DII scores and BMI. Seventh, participants in Project Viva were predominantly White and generally of higher socioeconomic status, potentially limiting generalizability to other cohorts.

## Conclusions

The findings of this study suggest that a proinflammatory diet in pregnancy is associated with greater adiposity accrual from childhood through adolescence. Furthermore, maternal depressive symptoms and racial and ethnic minority group and language status were associated with greater susceptibility to the adipogenic effects of a proinflammatory diet in pregnancy. Future studies investigating dietary interventions or metabolic control during pregnancy in offspring adiposity outcomes should evaluate the role of different stress phenotypes. Concurrently addressing maternal diet quality and well-defined stress measures in studies evaluating developmental origins of child obesity will most comprehensively inform practice and policy.

## Notes

Supplement 1. **eMethods.** Detailed Methods **eResults.** Detailed Results **eFigure 1.** Flow Diagram of Participants (n=1,060) for This Analysis From Project Viva, a Cohort Recruited From Boston, Massachusetts, Area in 1999-2002 **eTable 1.** Characteristics of Mother-Child Dyads Included vs Excluded From the Current Analysis in Project Viva **eTable 2.** Childhood Size and Adiposity Measures in Project Viva in Early Childhood, Midchildhood, and Early Adolescence by Quartile of Maternal DII in Pregnancy **eTable 3.** Associations of Prenatal Dietary Inflammatory Index Score With Adiposity Indices Across Early Childhood, Midchildhood, and Early Adolescence Visits and Interactions With Child Age Among Mother-Child Dyads in Project Viva **eFigure 2.** Associations of Prenatal DII With Adiposity Childhood Stratified by Each Subindex Theme of the SVI **eTable 4.** Associations of Prenatal Dietary Inflammatory Index Score With BMI z-Score Across Early Childhood, Midchildhood, and Early Adolescence Visits and Interactions With Child Age Using Imputed Dataset for n=1285 Dyads **eTable 5.** Joint Associations of Prenatal Dietary Inflammatory Index and Edinburgh Postnatal Depression Scale (EPDS) During Pregnancy With BMI z-Score and Interactions With Child Age Using Imputed Dataset for n=1285 Dyads **eTable 6.** Associations of Pregnancy DII Score With BMI z-Score Across Early Childhood, Midchildhood, and Early Adolescence, and Interactions With Child Age, Stratified by Maternal SVI Status in Pregnancy Using Imputed Dataset for n=1285 Dyads
eReferences

Supplement 2.
Data Sharing Statement
