# Supplementary material for: The Role of Prenatal Psychosocial Stress in the Associations of a Proinflammatory Diet in Pregnancy With Child Adiposity and Growth Trajectories
Source: JAMA Netw Open. 2023 Jan 20;6(1):e2251367. doi: 10.1001/jamanetworkopen.2022.51367 (PMC9860526; doi:10.1001/jamanetworkopen.2022.51367)
Supplement: Supplement 1. — eMethods. Detailed Methods eResults. Detailed Results eFigure 1. Flow Diagram of Participants (n=1,060) for This Analysis From Project Viva, a Cohort Recruited From Boston, Massachusetts, Area in 1999-2002 eTable 1. Characteristics of Mother-Child Dyads Included vs Excluded From the Current Analysis in Project Viva eTable 2. Childhood Size and Adiposity Measures in Project Viva in Early Childhood, Midchildhood, and Early Adolescence by Quartile of Maternal DII in Pregnancy eTable 3. Associations of Prenatal Dietary Inflammatory Index Score With Adiposity Indices Across Early Childhood, Midchildhood, and Early Adolescence Visits and Interactions With Child Age Among Mother-Child Dyads in Project Viva eFigure 2. Associations of Prenatal DII With Adiposity Childhood Stratified by Each Subindex Theme of the SVI eTable 4. Associations of Prenatal Dietary Inflammatory Index Score With BMI z-Score Across Early Childhood, Midchildhood, and Early Adolescence Visits and Interactions With Child Age Using Imputed Dataset for n=1285 Dyads eTable 5. Joint Associations of Prenatal Dietary Inflammatory Index and Edinburgh Postnatal Depression Scale (EPDS) During Pregnancy With BMI z-Score and Interactions With Child Age Using Imputed Dataset for n=1285 Dyads eTable 6. Associations of Pregnancy DII Score With BMI z-Score Across Early Childhood, Midchildhood, and Early Adolescence, and Interactions With Child Age, Stratified by Maternal SVI Status in Pregnancy Using Imputed Dataset for n=1285 Dyads eReferences [file jamanetwopen-e2251367-s001.pdf]

# Supplementary Online Content

Monthé-Drèze C, Aris IM, Rifas-Shiman SL, et al. The role of prenatal psychosocial stress in the associations of a proinflammatory diet in pregnancy with child adiposity and growth trajectories. *JAMA Netw Open*. 2023;6(1):e2251367. doi:10.1001/jamanetworkopen.2022.51367


**eMethods.** Detailed Methods

**eResults.** Detailed Results

**eFigure 1.** Flow Diagram of Participants (n=1,060) for This Analysis From Project Viva, a Cohort Recruited From Boston, Massachusetts, Area in 1999-2002

**eTable 1.** Characteristics of Mother-Child Dyads Included vs Excluded From the

Current Analysis in Project Viva

**eTable 2.** Childhood Size and Adiposity Measures in Project Viva in Early Childhood, Midchildhood, and Early Adolescence by Quartile of Maternal DII in Pregnancy

**eTable 3.** Associations of Prenatal Dietary Inflammatory Index Score With Adiposity Indices Across Early Childhood, Midchildhood, and Early Adolescence Visits and Interactions With Child Age Among Mother-Child Dyads in Project Viva

**eFigure 2.** Associations of Prenatal DII With Adiposity Childhood Stratified by Each Subindex Theme of the SVI

**eTable 4.** Associations of Prenatal Dietary Inflammatory Index Score With BMI z-Score Across Early Childhood, Midchildhood, and Early Adolescence Visits and Interactions With Child Age Using Imputed Dataset for n=1285 Dyads

**eTable 5.** Joint Associations of Prenatal Dietary Inflammatory Index and Edinburgh Postnatal Depression Scale (EPDS) During Pregnancy With BMI z-Score and Interactions With Child Age Using Imputed Dataset for n=1285 Dyads

**eTable 6.** Associations of Pregnancy DII Score With BMI z-Score Across Early Childhood, Midchildhood, and Early Adolescence, and Interactions With Child Age, Stratified by Maternal SVI Status in Pregnancy Using Imputed Dataset for n=1285 Dyads

**eReferences**


This supplementary material has been provided by the authors to give readers additional information about their work.

© 2023 Monthé-Drèze C et al. *JAMA Network Open.*

**eMethods.** Detailed Methods

Study Design

Project Viva is an ongoing prospective cohort study of prenatal and perinatal influences on maternal and child health. We recruited pregnant women at their initial prenatal visit at eight urban and suburban practices of a multi-specialty group practice in eastern Massachusetts between April 1999 and July 2002 (1). Exclusion criteria included multiple gestation, inability to answer questions in English, gestational age ≥22 weeks at recruitment and plans to move away before delivery. Trained research assistants completed in-person visits with mothers during pregnancy in the late first (median 9.9 weeks of gestation) and second (median 27.9 weeks) trimesters. Research assistants saw mothers and children in the hospital during the delivery admission and during early childhood (median 3.2 years; 2003-2006), mid-childhood (median 7.7 years; 2007-2010) and early adolescence (median 12.9 years; 2013-2016). At each visit, Mothers provided written informed consent, and children provided verbal assent at follow-up visits.

Dietary inflammation in pregnancy

Mothers completed self-administered FFQs at the first (median 9.9 wk of gestation) and second (median 27.9 wk of gestation) study visits in pregnancy. To obtain estimates of nutrients, we used the Harvard nutrient composition database, which is based primarily on USDA publications, as previously described (2,3). Resultant dietary data were used to calculate DII scores for each mother. The DII is a validated literature-based and population-based measure that has been developed to characterize and quantify the cumulative inflammatory potential of an individual's diet (4). The DII is not a dietary pattern, but an assessment of the pro- or anti-inflammatory potential of any diet. A detailed procedure for DII estimations in this cohort has been described previously (5). The 28 dietary parameters used for DII calculation are energy, carbohydrate, protein, fat, alcohol, fiber, cholesterol, SFAs, MUFAs, PUFAs, n–3 and n–6 FAs, *trans*-fat, niacin, thiamin, riboflavin, vitamin B-12, vitamin B-6, iron, magnesium, zinc, selenium, vitamin A, vitamin C, vitamin D, vitamin E, folic acid, and β-carotene. The DII ranges from −9 to +8 units, and a higher (i.e., more positive) DII score indicates a more proinflammatory diet, whereas a more negative score represents a more anti-inflammatory diet.

Social Vulnerability Index:

The social vulnerability index (SVI) is a validated index that was developed by the Center for Disease Control and Prevention to identify at-risk populations who are especially vulnerable in the face of a stressor or during public health emergencies (6,7). As detailed previously, the SVI is derived from a set of 15 US census community-level factors divided

© 2023 Monthé-Drèze C et al. *JAMA Network Open.*

into 4 themes or sub-indices based on the US Census 2000: (1) socioeconomic status, which includes the following social factors: below poverty, unemployment, median income, and no high school diploma; (2) household composition and disability, which includes the following factors: age 65 years or older, age 17 years or younger, disability status, and single-parent household; (3) racial/ethnic minority and language status, which includes the following factors: minority status, speaks English less than well; and (4) housing and transportation type , which includes the following factors: multiunit structure, mobile home, crowding, group quarter, and no vehicle (8,9). We conceptualized these socially based factors as chronic stressors that may play a critical role in creating vulnerability to dietary exposure in pregnancy. We geocoded each participant's residential address obtained in early pregnancy using ArcGIS (Esri, Redlands, CA) and linked the resultant census tract location for each participant to census tract-level SVI data for the year 2000 (the year closest to the pregnancy study visit). For each census tract, percentile ranks are generated for the 15 individual variables, the 4 themes, and the overall tract ranking. We used the overall and individual themes ranking as indicators for social vulnerability, and based on prior literature, we considered the top quartile to be the most vulnerable.

Covariates

Mothers reported their age, height, pre-pregnancy weight, education level, race and ethnicity (from the following options: non-Hispanic White, non-Hispanic Black, Hispanic, Asian, and others), parity, household income, and smoking status via interviews and questionnaires at enrollment, and we calculated pre-pregnancy BMI. For race and ethnicity, if a participant chose more than 1 racial or ethnic group, we classified them in the "other" category, which also included American Indian or Alaskan Native. We calculated gestational age (GA) at birth based on established methods (10).

Statistical analysis: Methods for multiple imputation

We used multiple imputation by chained equations (11) to impute values for missing covariates. Briefly, multiple imputation generates multiple predictions for each missing value derived from distributions of and associations among observed variables in the data set. Using multiple plausible values would not only quantify the uncertainty in the imputations but also yield more accurate SEs, reducing the likelihood of spurious results (12). We chose this method over single-value imputation methods, which often fail to account for uncertainty in imputing missing values, do not use all available information, can introduce bias, and artificially increase precision (13). We generated 50 imputed data sets for all 2,128 Project Viva children. The imputation model included the primary outcome (ie., BMI z-score) as well as all exposures and covariates under study. We combined imputed datasets using MI ESTIMATE in Stata after excluding those participants who did not satisfy the inclusion criteria for this study (n=843, eFigure 1 in the supplement), leaving a sample size of n=1285 for imputed data sets. To assess the robustness of our study findings, we conducted sensitivity analyses

© 2023 Monthé-Drèze C et al. *JAMA Network Open.*

by repeating all analyses with our primary outcome BMI z-score using imputed data from these 1285 dyads and compared results to our complete case analysis.

© 2023 Monthé-Drèze C et al. *JAMA Network Open.*

**eResults.** Detailed Results

Study population:

Of 2,128 mother-child dyads, there were 1,886 (88.6%) dyads with available FFQ data at any point in pregnancy and without type 1 or 2 diabetes melitus in pregnancy. We excluded n=136 children born preterm (< 37 weeks), n=465 children with no outcome data at any timepoints and n=225 participants with missing covariate data. The final analytical sample included 1,060 children who had ≥1 outcome data available during early childhood (N=961; mean (SD) age 3.3 (0.3) years), mid-childhood (N=812; mean (SD) 7.9 (0.8) years) or early adolescence (N=773; mean (SD) age 13.2 (0.9) years) study visits (**eFigure 1**).

© 2023 Monthé-Drèze C et al. *JAMA Network Open.*

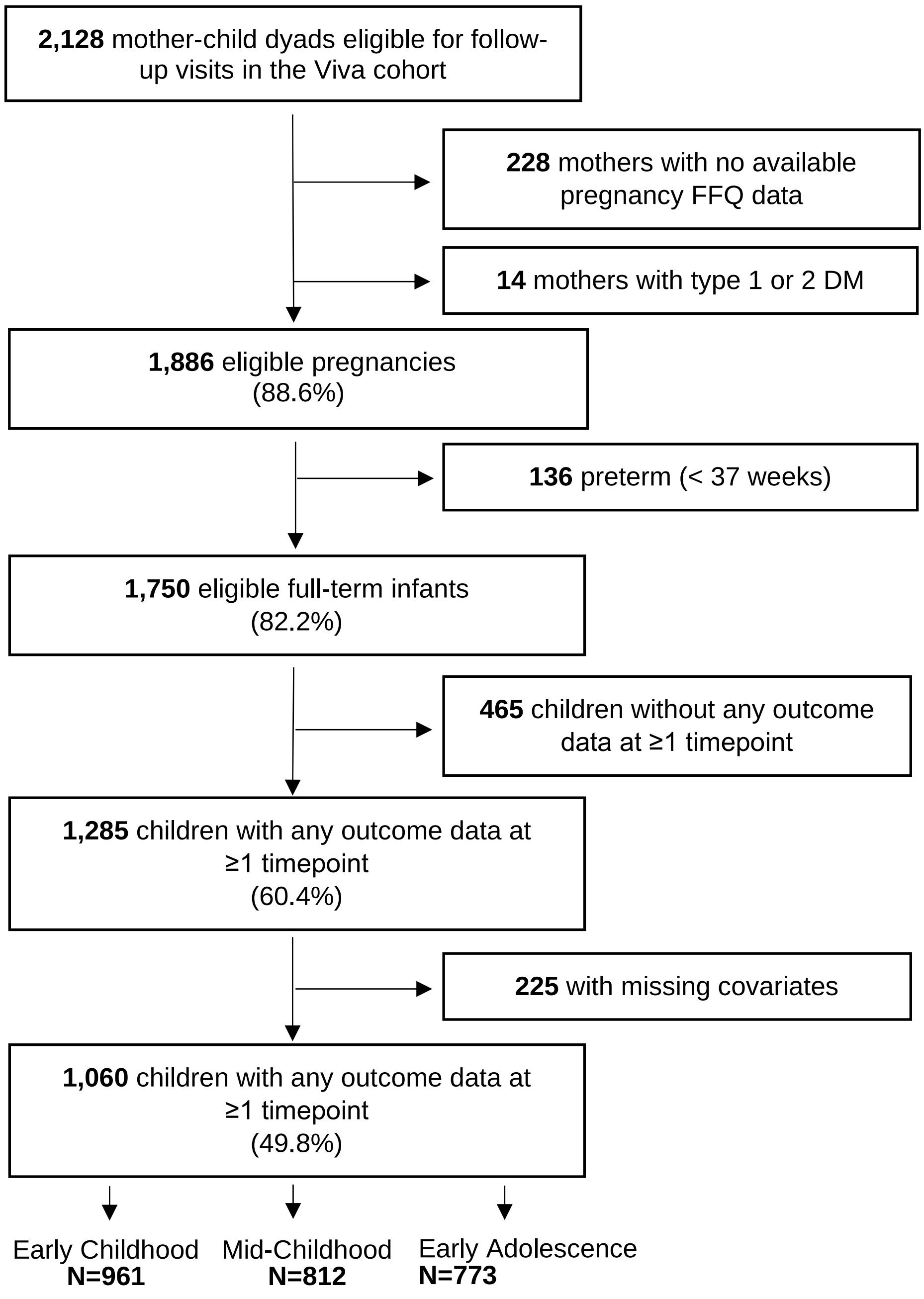


**eFigure 1. Flow diagram of participants (n=1,060) for this analysis from Project Viva, a cohort recruited from Boston, Massachusetts area in 1999-2002. Abbreviation: FFQ, food frequency questionnaire; DM Diabetes mellitus**

© 2023 Monthé-Drèze C et al. *JAMA Network Open.*

**eTable 1. Characteristics of mother-child dyads included vs. excluded from the current analysis in Project Viva**

| **Maternal Characteristics** | **Included n=1,060** | **Excluded n=1068** | ***P* value** |
|---|---|---|---|
| Age, mean (SD), y | 32.6 (4.6) | 31.0 (5.7) | <0.001 |
| DII, mean (SD), units | -2.7 (1.3) | -2.4 (1.5) | <0.001 |
| Pre-pregnancy BMI, mean (SD), $kg/m^2$ | 24.4 (4.9) | 25.4 (6.0) | <0.001 |
| Education, No (%) | | | <0.001 |
| Not a college graduate | 268 (25) | 476 (46) | |
| College graduate | 792 (75) | 568 (54) | |
| Race/ethnicity, No (%) | | | <0.001 |
| Asian or Pacific Islander | 54 (5) | 66 (6) | |
| Hispanics | 51 (5) | 103 (10) | |
| Non-Hispanic Black | 106 (10) | 242 (23) | |
| Non-Hispanic White | 811 (77) | 588 (56) | |
| Other [a] | 38 (3) | 45 (4) | |
| Nulliparous, No (%) | | | 0.24 |
| No | 540 (51) | 571 (53) | |
| Yes | 520 (49) | 497 (47) | |
| Household income >$70,000/year, No (%) | | | <0.001 |
| No | 369 (35) | 359 (44) | |
| Yes | 691 (65) | 455 (56) | |
| Pregnancy smoking status, No (%) | | | <0.001 |
| Never | 724 (68) | 719 (69) | |
| Former | 233 (22) | 165 (16) | |
| Smoked during pregnancy | 103 (10) | 163 (15) | |
| SVI | | | |
| Overall percentile ranking, mean (SD), %ile | 38 (27) | 47 (30) | <0.001 |
| Depressive symptoms, No (%) | | | 0.20 |
| No (EPDS <13) | 972 (92) | 511 (90) | |
| Yes (EPDS ≥13) | 88 (8) | 58 (10) | |
| **Child Characteristics** | | | |
| Sex, No (%) | | | 0.02 |
| Male | 520 (49) | 576 (54) | |
| Female | 540 (51) | 492 (46) | |

Values are expressed as n (%) for categorical variables (compared using the chi-square test) and mean ± SD for continuous variables (compared using two-sample t-test). **Abbreviations:** BMI, Body mass index; DII, Dietary inflammatory index; EPDS: Edinburgh postnatal depression scale, SVI, Social Vulnerability Index
[a] Other category included American Indian or Alaskan Native, more than one race and ethnicity

© 2023 Monthé-Drèze C et al. *JAMA Network Open.*

**eTable 2. Childhood size and adiposity measures in Project Viva in early childhood, midchildhood, and early adolescence by quartile of maternal DII in pregnancy[a]**

| | | | DII Quartile (Average first and second trimesters) | | | | |
|---|---|---|---|---|---|---|---|
| | n | All | Q1 | Q2 | Q3 | Q4 | *P value* |
| **Anthropometry Measures** | | | | | | | |
| ***Overall Adiposity*** BMI-z, mean (SD), units | | | | | | | |
| Early childhood | 951 | 0.7 (1.0) | 0.6 (0.9) | 0.7 (1.0) | 0.8 (1.0) | 0.9 (1.0) | 0.09 |
| Mid-childhood | 807 | 0.5 (1.1) | 0.4 (1.2) | 5.5 (1.1) | 0.5 (1.0) | 0.8 (1.1) | 0.001 |
| Early adolescence | 770 | 0.5 (1.2) | 0.2 (1.21) | 0.5 (1.1) | 0.5 (1.2) | 0.7 (1.2) | <0.001 |
| SS+TR, mean (SD), mm | | | | | | | |
| Early childhood | 931 | 16.7 (4.2) | 16.6 (4.0) | 17.1 (4.2) | 16.6 (4.3) | 16.7 (4.3) | 0.37 |
| Mid-childhood | 808 | 19.3 (8.9) | 18.7 (8.6) | 18.5 (7.9) | 18.6 (8.2) | 21.7 (10.7) | <0.001 |
| Early adolescence | 771 | 27.6 (13.1) | 26.2 (12.0) | 27.7 (12.6) | 27.1 (12.4) | 30.1 (15.3) | 0.03 |
| ***Central Adiposity*** | | | | | | | |
| WC, mean (SD), cm | | | | | | | |
| Early childhood | 958 | 51.4 (3.6) | 51.1 (3.3) | 51.4 (3.2) | 51.4 (3.8) | 51.5 (4.3) | 0.67 |
| Mid-childhood | 811 | 59.5 (7.6) | 59.1 (7.4) | 58.8 (7.2) | 59.3 (6.6) | 61.2 (9.0) | 0.01 |
| Early adolescence | 773 | 72.6 (11.3) | 71.5 (10.9) | 72.8 (11.4) | 72.1 (10.1) | 74.5 (12.7) | 0.07 |
| SS/TR ratio, mean (SD), % | | | | | | | |
| Early childhood | 931 | 63.9 (15.4) | 65.3 (16.6) | 62.9 (15.0) | 63.1 (15.6) | 64.3 (14.2) | 0.27 |
| Mid-childhood | 808 | 69.4 (17.5) | 69.7 (18.7) | 67.7 (16.4) | 68.9 (15.3) | 71.4 (19.4) | 0.25 |
| Early adolescence | 771 | 76.9 (22.9) | 75.9 (20.2) | 76.3 (22.5) | 75.5 (22.7) | 80.5 (26.5) | 0.14 |
| **Body Composition Measures** | | | | | | | |
| ***Overall Adiposity*** | | | | | | | |
| BIA FMI, mean (SD), kg/m$^2$ | | | | | | | |
| Mid-childhood | 806 | 3.8 (1.9) | 3.2 (1.9) | 3.3 (1.8) | 3.3 (1.7) | 3.9 (2.1) | 0.002 |
| Early adolescence | 762 | 4.8 (3.2) | 4.4 (2.9) | 4.7 (3.1) | 4.4 (2.9) | 5.6 (3.8) | <0.001 |
| BIA Fat %, mean (SD), % | | | | | | | |
| Mid-childhood | 806 | 18.8 (6.8) | 18.2 (7.0) | 18.5 (6.4) | 18.2 (6.1) | 20.7 (7.7) | <0.001 |
| Early Adolescence | 762 | 21.4 (9.9) | 20.6 (9.7) | 21.4 (9.7) | 20.0 (9.3) | 24.0 (10.6) | <0.001 |
| DXA FMI, mean (SD), kg/m$^2$ | | | | | | | |
| Mid-childhood | 629 | 4.3 (1.7) | 4.2 (1.8) | 4.2 (1.6) | 4.1 (1.5) | 4.8 (2.0) | 0.002 |
| Early adolescence | 546 | 6.2 (2.9) | 6.0 (2.7) | 6.1 (2.8) | 6.0 (2.7) | 6.8 (3.4) | 0.08 |
| DXA Fat %, mean (SD), % | | | | | | | |
| Mid-childhood | 629 | 24.4 (6.0) | 24.3 (6.0) | 24.1 (5.8) | 23.6 (5.8) | 25.8 ( 6.4) | 0.01 |
| Early adolescence | 546 | 28.5 (7.3) | 28.4 (6.9) | 28.4 (7.5) | 27.7 (7.1) | 29.7 (7.6) | 0.15 |
| ***Central Adiposity*** DXA Trunk FMI, mean (SD), kg/m$^2$ | | | | | | | |
| Mid-childhood | 629 | 1.4 (0.8) | 1.4 (0.8) | 1.4 (0.7) | 1.3 (0.7) | 1.6 (0.4) | 0.005 |
| Early adolescence | 546 | 2.4 (1.4) | 2.2 (1.2) | 2.3 (1.4) | 2.2 (1.3) | 2.6 (1.7) | 0.07 |

[a] Values are expressed as mean (SD) and compared using the ANOVA test. Early childhood: mean (SD) age = 3.3 (0.3) y, (n=961); Mid childhood: mean (SD) age = 7.9 (0.8) y, (n=812); Early adolescence: mean (SD) age = 13.2 (0.9) y, (n=773). N=854, N=846, N=853, N=658, and N=471 children

© 2023 Monthé-Drèze C et al. *JAMA Network Open.*

have BMI z-score, WC, skinfolds, BIA and DXA data, respectively, at ≥2 visits. **Abbreviations:** BMI-z, WHO age- and sex-standardized BMI score, DII, Dietary inflammatory index; BIA, Bioelectrical impedance analysis; DXA, dual x-ray absorptiometry, FMI, Fat Mass Index; SS, subscapular skinfold; TR, triceps skinfold WC, waist circumference

© 2023 Monthé-Drèze C et al. *JAMA Network Open.*

**eTable 3. Associations of prenatal dietary inflammatory index score with adiposity indices across early childhood, midchildhood, and early adolescence visits and interactions with child age among mother-child dyads in Project Viva**

| | DII Quartile | Diet*Age Interaction β (95% CI) [a] | 3 years β (95% CI) [b] | 8 years β (95% CI) [c] | 13 years β (95% CI) [d] |
|---|---|---|---|---|---|
| **Overall Adiposity** | **Anthropometry Measures** | | | | |
| BMI z, SD units (n=1,055) | Q1 | Ref | Ref | Ref | Ref |
| | Q2 | 0.02 (-0.00 to 0.04) | -0.05 (-0.21 to 0.12) | 0.05 (-0.11 to 0.21) | 0.15 (-0.06 to 0.35) |
| | Q3 | 0.01 (-0.01 to 0.03) | -0.00 (-0.17 to 0.17) | 0.04 (-0.11 to 0.20) | 0.09 (-0.12 to 0.30) |
| | Q4 | 0.03 (0.01-0.05) | 0.05 (-0.13 to 0.24) | 0.21 (0.04-0.38) | 0.36 (0.14-0.59) |
| *P*-trend [e] | | 0.02 | 0.53 | 0.04 | 0.006 |
| SS+TR, mm | Q1 | Ref | Ref | Ref | Ref |
| (n=1,049) | Q2 | 0.04 (-0.19 to 0.27) | 0.40 (-0.39 to 1.20) | 0.61 (-0.64 to 1.87) | 0.82 (-1.47 to 3.11) |
| | Q3<br>Q4 | 0.05 (-0.18 to 0.28)<br>0.40 (0.16-0.65) | -0.06 (-0.87 to 0.75)<br>-0.05 (-0.94 to 0.85) | 0.20 (-1.06 to 1.46)<br>1.96 (0.61-3.31) | 0.46 (-1.82 to 2.74)<br>3.97 (1.55-6.39) |
| *P*-trend [e] | | 0.004 | 0.714 | 0.017 | 0.005 |
| **Central Adiposity** | | | | | |
| WC, cm (n=1,054) | Q1 | Ref | Ref | Ref | Ref |
| | Q2 | 0.05 (-0.13 to 0.24) | 0.11 (-0.55 to 0.77) | 0.37 (-0.71 to 1.45) | 0.64 (-1.28 to 2.55) |
| | Q3 | 0.00 (-0.1 to 0.19) | 0.22 (-0.45 to 0.89) | 0.23 (-0.86 to 1.31) | 0.23 (-1.67 to 2.14) |
| | Q4 | 0.30 (0.10-0.50) | 0.24 (-0.50 to 0.98) | 1.73 (0.57-2.89) | 3.22 (1.19-5.24) |
| *P*-trend [e] | | 0.02 | 0.47 | 0.01 | 0.008 |
| SS/TR ratio, % (n=1,049) | Q1 | Ref | Ref | Ref | Ref |
| | Q2<br>Q3 | 0.25 (-0.21 to 0.71)<br>0.19 (-0.27 to 0.65) | -2.01 (-4.63 to 0.61)<br>-2.71 (-5.36 to -0.05) | -0.74 (-3.17 to 1.68)<br>-1.74 (-4.18 to 0.70) | 0.52 (-3.42 to 4.46)<br>-0.78 (-4.70 to 3.14) |
| | Q4 | 0.61 (0.12-1.10) | -3.11 (-6.03 to -0.20) | -0.06 (-2.72 to 2.59) | 2.99 (-1.20 to 7.17) |
| *P*-trend [e] | | 0.03 | 0.03 | 0.67 | 0.32 |
| **Overall Adiposity** | | **Body Composition Measures** | | | |
| | | | | | |
| BIA FMI, kg/m$^2$ | Q1 | Ref | N/A | Ref | Ref |
| n=908 | Q2<br>Q3 | 0.03 (-0.05 to 0.11)<br>-0.04 (-0.12 to 0.04) | | 0.09 (-0.23 to 0.42)<br>-0.10 (-0.44 to 0.23) | 0.25 (-0.31 to 0.80)<br>-0.32 (-0.87 to 0.23) |
| | Q4 | 0.11 (0.03-0.19) | | 0.39 (0.02-0.75) | 0.94 (0.35-1.53) |
| *P*-trend [e] | | 0.08 | | 0.15 | 0.04 |
| BIA fat % | Q1 | Ref | N/A | Ref | Ref |
| n= 908 | Q2<br>Q3 | 0.05 (-0.21 to 0.31) | | 0.37 (-0.80 to 1.54) | 0.62 (-1.02 to 2.27) |

© 2023 Monthé-Drèze C et al. *JAMA Network Open.*

| | | | | | |
|---|---|---|---|---|---|
| | | -0.20 (-0.46 to 0.06) | | -0.47 (-1.67 to 0.72) | -1.48 (-3.12 to 0.15) |
| | Q4 | 0.21 (-0.07 to 0.49) | | 1.17 (-0.14 to 2.47) | 2.21 (0.46-3.97) |
| *P*-trend [e] | | 0.53 | | 0.27 | 0.19 |
| DXA total FMI, kg/$m^2$ | Q1 | Ref | N/A | Ref | Ref |
| n= 704 | Q2 | 0.00 (-0.08, 0.09) | | -0.05 (-0.39 to 0.29) | -0.02 (-0.62 to 0.58) |
| | Q3 | 0.01 (-0.07 to 0.09) | | -0.14 (-0.49 to 0.21) | -0.07 (-0.67 to 0.52) |
| | Q4 | 0.09 (0.00-0.17) | | 0.38 (0.01-0.76) | 0.83 (0.20-1.46) |
| *P*-trend [e] | | 0.05 | | 0.12 | 0.03 |
| DXA fat % | Q1 | Ref | N/A | Ref | Ref |
| n= 704 | Q2 | -0.04 (-0.26 to 0.18) | | -0.13 (-1.24 to 0.98) | -0.33 (-1.84 to 1.19) |
| | Q3 | -0.00 (-0.22 to 0.21) | | -0.71 (-1.85 to 0.43) | -0.73 (-2.24 to 0.78) |
| | Q4 | 0.08 (-0.15 to 0.31) | | 0.83 (-0.39 to 2.06) | 1.24 (-0.37 to 2.85) |
| *P*-trend [e] | | 0.47 | | 0.42 | 0.26 |
| **Central Adiposity** | | | | | |
| DXA Trunk FMI | Q1 | Ref | N/A | Ref | Ref |
| n=704 | Q2 | 0.01 (-0.04 to 0.05) | | -0.03 (-0.18 to 0.12) | -0.00 (-0.29 to 0.28) |
| | Q3 | 0.00 (-0.04 to 0.04) | | -0.07 (-0.22 to 0.09) | -0.04 (-0.33 to 0.24) |
| | Q4 | 0.05 (0.01-0.09) | | 0.15 (-0.01 to 0.32) | 0.39 (0.09-0.70) |
| *P*-trend [e] | | 0.04 | | 0.17 | 0.03 |

[a] Linear mixed effects models were used to estimate mean differences in rates of gain (per year) for each child adiposity measure by quartile of maternal DII among mother-child pairs. Maternal DII was included as a fixed effect and as an interaction with age and adjusted for child age and sex (except for the BMI-z outcome); maternal age at enrollment, race/ethnicity, education, parity, household income, pregnancy smoking status, and pre-pregnancy BMI. **Abbreviations:** BMI-z, WHO age- and sex-standardized BMI score, DII, Dietary inflammatory index; BIA, Bioelectrical impedance analysis; DXA, dual x-ray absorptiometry; FMI, Fat Mass Index; SS, subscapular skinfold; TR, triceps skinfold; WC, waist circumference.

[b,c,d] Post-estimation margins command in Stata were used to estimate mean differences for each child adiposity measure by quartile of maternal DII at 3 (early childhood), 8 (mid-childhood), and 13 (early adolescence) years old following the adjusted linear mixed model above.

[e] *P*-trend obtained from linear mixed-effects models constructed as above using the 4-category DII variable as a continuous variable

© 2023 Monthé-Drèze C et al. *JAMA Network Open.*

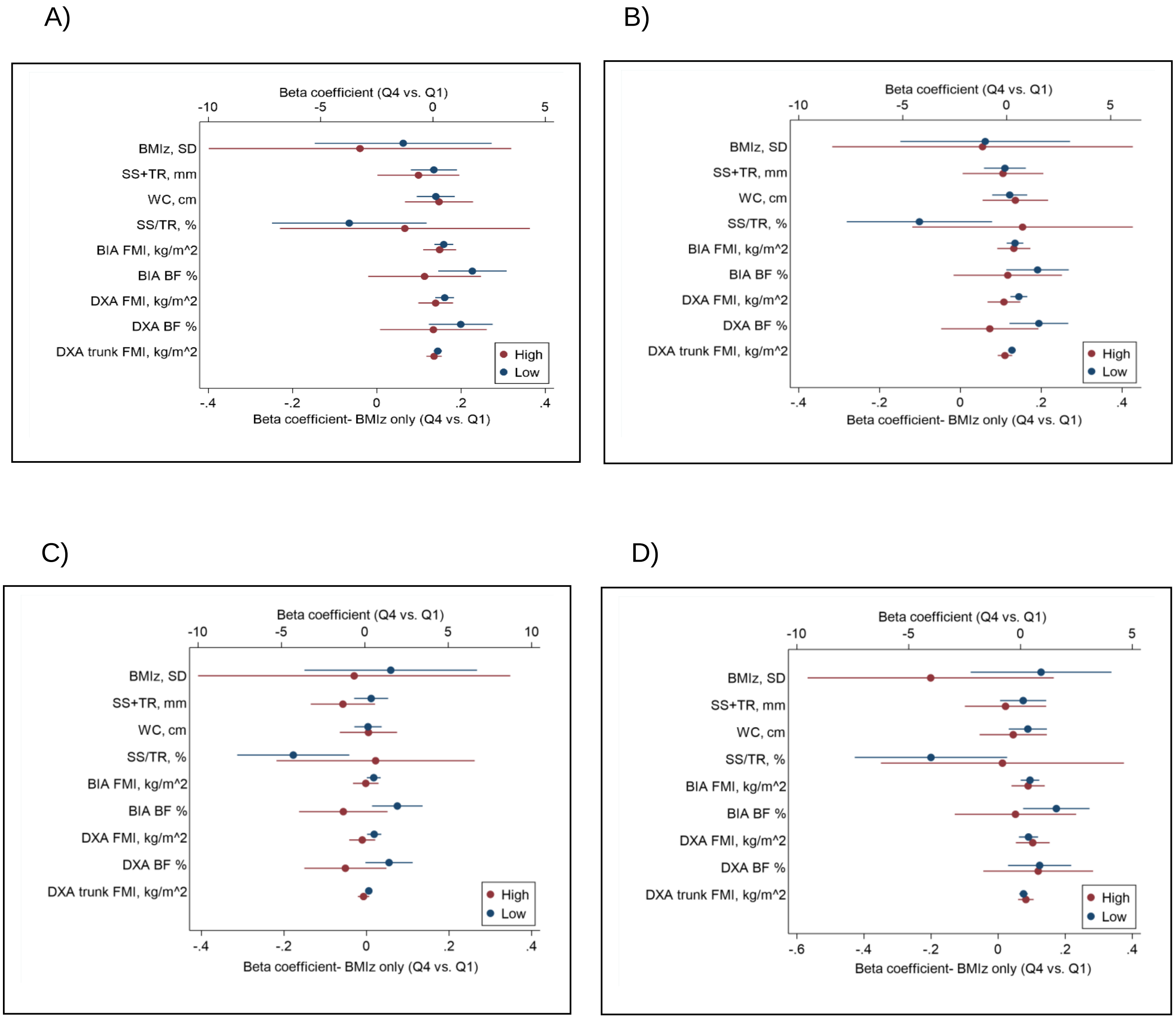


**eFigure 2. Associations of prenatal DII with adiposity childhood stratified by each sub-index theme of the SVI. Results displayed are beta coefficient with 95% CI from linear mixed model analyses with prenatal DII as a fixed effect and as an interaction with age and adjusted for child age and sex (except for the BMI-z outcome), confounders (maternal age at enrollment, race and ethnicity, education, parity, household income, pregnancy smoking status, and pre-pregnancy BMI), stratified by each SVI sub-index category (high, or most vulnerable: > 75%ile; Low, or less vulnerable: < 75%ile) A. Socioeconomic Sub-Index; B. Household/Disability Sub-Index; C. Minority/language Sub-Index; D. Housing/Transportation Sub-Index. Abbreviations*:* BMI-z, WHO age- and sex-standardized body mass index score, Dietary inflammatory index; FMI, Fat mass index; SS, subscapular skinfold; SVI: Social Vulnerability Index; TR, triceps skinfold thickness; WC, waist circumference**

© 2023 Monthé-Drèze C et al. *JAMA Network Open.*

**eTable 4. Associations of prenatal dietary inflammatory index score with BMI z-score across early childhood, midchildhood, and early adolescence visits and interactions with child age using imputed dataset for n=1285 dyads [a]**

| | DII Quartile | Diet*Age Interaction β (95% CI) [a] | 3 years β (95% CI) [b] | 8 years β (95% CI) [c] | 13 years β (95% CI) [d] |
|---|---|---|---|---|---|
| **Overall Adiposity** | **Anthropometry Measures** | | | | |
| BMI-z, SD units N=1285 | Q1 | Ref | Ref | Ref | Ref |
| | Q2 | 0.02 (-0.00 to 0.04) | -0.03 (-0.19 to 0.13) | 0.07 (-0.07 to 0.21) | 0.17 (-0.02 to 0.37) |
| | Q3 | 0.01 (-0.01 to 0.04) | -0.04 (-0.20 to 0.12) | 0.03 (-0.11 to 0.16) | 0.10 (-0.09 to 0.28) |
| | Q4 | 0.03 (0.01-0.05) | -0.00 (-0.17 to 0.17) | 0.16 (0.01-0.31) | 0.32 (0.12-0.52) |
| *P*-trend [e] | | 0.01 | 0.92 | 0.08 | 0.006 |

[a] Linear mixed effects models were used to estimate mean differences in rates of gain (per year) for each child adiposity measure by quartile of maternal DII among mother-child pairs. Maternal DII was included as a fixed effect and as an interaction with age and adjusted for child age and sex (except for the BMI-z outcome); maternal age at enrollment, race/ethnicity, education, parity, household income, pregnancy smoking status, and pre-pregnancy BMI. **Abbreviations:** BMI-z, WHO age- and sex-standardized BMI score, DII, Dietary inflammatory index; SD, Standard deviation.

[b,c,d] Post-estimation margins command in Stata were used to estimate mean differences for each child adiposity measure by quartile of maternal DII at 3 (early childhood), 8 (mid-childhood), and 13 (early adolescence) years old following the adjusted linear mixed model above.

[e] *P*-trend obtained from linear mixed-effects models constructed as above using the 4-category DII variable as a continuous variable

© 2023 Monthé-Drèze C et al. *JAMA Network Open.*

**eTable 5. Joint Associations of prenatal dietary inflammatory index and Edinburgh Postnatal Depression Scale (EPDS) during pregnancy with BMI z-score and interactions with child age using imputed dataset for n=1285 dyads [a]**

| | DII Quartile | EPDS Scores Category | | EPDS*DII Interaction β (95% CI) | EPDS*DII*Age* Interaction β (95% CI) |
|---|---|---|---|---|---|
| | | Anthropometry Measures | | | |
| **Overall Adiposity** | Q1 | Low | | Ref | Ref |
| BMI-z, SD units N=1285 | Q2 | High | | 0.12 (-0.45 to 0.69) | 0.00 (-0.08 to 0.09) |
| | Q3 | High | | 0.13 (-0.43 to 0.69) | -0.03 (-0.11 to 0.05) |
| | Q4 | High | | 0.18 (-0.34 to 0.70) | -0.01 (-0.08 to 0.07) |
| *P*-trend [b] | | | | 0.50 | 0.75 |

[a] Linear mixed effects models were used to estimate joint associations of maternal DII and EPDS among mother-child pairs with child adiposity. Maternal DII quartile category and EPDS category (positive vs. negative screen) were included as a fixed effect and as an interaction with age. Model also included DII*EPDS and DII*EPDS*Age interaction terms. Model adjusted for child age and sex (except for the BMI-z outcome), maternal age at enrollment, race/ethnicity, education, parity, household income, pregnancy smoking status, and pre-pregnancy BMI. **Abbreviations**: BMI-z, WHO age- and sex-standardized BMI score; DII, Dietary inflammatory index; EPDS, Edinburgh Postpartum Depression Scale; SD, Standard deviation.

[b] *P*-trend obtained from linear mixed-effects models constructed as above using the 4-category DII variable as a continuous variable

© 2023 Monthé-Drèze C et al. *JAMA Network Open.*

**eTable 6. Associations of pregnancy DII score with BMI z-score across early childhood, mid-childhood, and early adolescence, and interactions with child age, stratified by maternal SVI status in pregnancy using imputed dataset for n=1285 dyads [a]**

| | | | High SVI (Most vulnerable, >75%ile) | | | | Low SVI (Least vulnerable, <75%ile) | |
|---|---|---|---|---|---|---|---|---|
| | **DII Quartile** | **n** | **Main Effect β (95% CI)** | **Diet*Age Interaction β (95% CI)** | **DII Quartile** | **n** | **Main effect β (95% CI)** | **Diet*Age Interaction β (95% CI)** |
| **Overall Adiposity** | | | **Anthropometric Measures** | | | | | |
| BMI-z, SD units N=1285 | Q1 | | Ref | Ref | Q1 | | Ref | Ref |
| | Q2 | | 0.14 (-0.25 to 0.54) | 0.03 (-0.02 to 0.08) | Q2 | | -0.08 (-0.26 to 0.09) | 0.02 (-0.01 to 0.04) |
| | Q3 | | -0.26 (-0.63 to 0.10) | 0.04 (-0.00 to 0.09) | Q3 | | 0.01 (-0.17 to 0.19) | 0.00 (-0.02 to 0.03) |
| | Q4 | | -0.18 (-0.53 to 0.17) | 0.04 (-0.00 to 0.09) | Q4 | | 0.06 (-0.13 to 0.25) | 0.03 (0.00-0.05) |
| *P*-trend [b] | | 326 | 0.13 | 0.05 | | 959 | 0.41 | 0.14 |

[a] Linear mixed effects models were used to estimate mean differences in each adiposity measure by quartile of maternal DII. We included quartile of maternal DII category as a fixed effect and as an interaction with age and adjusted for child age and sex (except for the BMI-z outcome); maternal age at enrollment, race/ethnicity, education, parity, household income, pregnancy smoking status, and pre-pregnancy BMI. **Abbreviations**: BMI-z, WHO age- and sex-standardized BMI score; SVI: Social Vulnerability Index; SD, Standard deviation.

[b] *P*-trend obtained from linear mixed-effects models constructed as above using the 4-category DII variable as a continuous variable

© 2023 Monthé-Drèze C et al. *JAMA Network Open.*

**eReferences**


1. Oken E, Baccarelli AA, Gold DR, Kleinman KP, Litonjua AA, De Meo D, Rich-Edwards JW, Rifas-Shiman SL, Sagiv S, Taveras EM, et al. Cohort profile: project viva. Int J Epidemiol 2015;44:37–48.

2. Oken E, Kleinman KP, Olsen SF, Rich-Edwards JW, Gillman MW. Associations of seafood and elongated n-3 fatty acid intake with fetal growth and length of gestation: results from a US pregnancy cohort. Am J Epidemiol 2004;160:774–83.

3. Gillman MW, Rifas-Shiman SL, Kleinman KP, Rich-Edwards JW, Lipshultz SE. Maternal Calcium Intake and Offspring Blood Pressure. Circulation NIH Public Access; 2004;110:1990.

4. Shivappa N, Steck SE, Hurley TG, Hussey JR, Hébert JR. Designing and developing a literature-derived, population-based dietary inflammatory index. Public Health Nutr 2014;17:1689–96.

5. Sen S, Rifas-Shiman SL, Shivappa N, Wirth MD, Hébert JR, Gold DR, Gillman MW, Oken E. Dietary Inflammatory Potential during Pregnancy Is Associated with Lower Fetal Growth and Breastfeeding Failure: Results from Project Viva. J Nutr 2016;146:728–36.

6. CDC Social Vulnerability Index 2018 - USA - Overview [Internet]. [cited 2022 Feb 5]. Available from: https://www.arcgis.com/home/item.html?id=cbd68d9887574a10bc89ea4efe2b8087

7. At A Glance: CDC/ATSDR Social Vulnerability Index | Place and Health | ATSDR [Internet]. 2021 [cited 2022 Feb 5]. Available from: https://www.atsdr.cdc.gov/placeandhealth/svi/at-a-glance_svi.html

8. Angelidou A, Sullivan K, Melvin PR, Shui JE, Goldfarb IT, Bartolome R, Chaudhary N, Vaidya R, Culic I, Singh R, et al. Association of Maternal Perinatal SARS-CoV-2 Infection With Neonatal Outcomes During the COVID-19 Pandemic in Massachusetts. JAMA Netw Open 2021;4:e217523.

9. Givens M, Teal EN, Patel V, Manuck TA. Preterm birth among pregnant women living in areas with high social vulnerability. Am J Obstet Gynecol MFM 2021;3:100414.

10. Aris IM, Rifas-Shiman SL, Li L-J, Kleinman K, Coull BA, Gold DR, Hivert M-F, Kramer MS, Oken E. Pre-, Perinatal, and Parental Predictors of Body Mass Index Trajectory Milestones. J Pediatr 2018;201:69-77.e8.

11. Buuren S van, Groothuis-Oudshoorn K. mice: Multivariate Imputation by Chained Equations in R. Journal of Statistical Software 2011;45:1–67.

12. Li P, Stuart EA, Allison DB. Multiple Imputation: A Flexible Tool for Handling Missing Data. JAMA 2015;314:1966–7.

13. Newgard CD, Lewis RJ. Missing Data: How to Best Account for What Is Not Known. JAMA 2015;314:940–1.


© 2023 Monthé-Drèze C et al. *JAMA Network Open.*
