# Supplementary material for: The Role of Prenatal Psychosocial Stress in the Associations of a Proinflammatory Diet in Pregnancy With Child Adiposity and Growth Trajectories
Source: JAMA Netw Open. 2023 Jan 20;6(1):e2251367. doi: 10.1001/jamanetworkopen.2022.51367 (PMC9860526; doi:10.1001/jamanetworkopen.2022.51367)
Supplement: Supplement 2. — Data Sharing Statement [file jamanetwopen-e2251367-s002.pdf]

Data Sharing Statement

Monthé-Drèze. The Role of Prenatal Psychosocial Stress in the Associations of a Proinflammatory Diet in Pregnancy With Child Adiposity and Growth Trajectories. *JAMA Netw Open*. Published January 20, 2023. doi:10.1001/jamanetworkopen.2022.51367

Data
Data available: Yes
Data types: Deidentified participant data, Data dictionary
How to access data: First author (cmonthedreze@bwh.harvard.edu) can be contacted for deidentified data requests.
When available: With publication

Supporting Documents
Document types: None

Additional Information
Who can access the data: Researchers where the proposed use of the data is approved as outline in the data sharing statement.
Types of analyses: Analyses will need to be for a specified purpose
Mechanisms of data availability: Analyses proposal requests will require review and approval by the Project Viva Co-I team and appropriate IRB approval. Once approved and a data access agreement has been executed, deidentified data generated from this research will be made available to affiliated investigators through secure database for the prespecified analysis.
